# Rolling bearing fault diagnosis in noisy environments using Channel-Time parallel attention networks

**DOI:** 10.1038/s41598-025-22683-y

**Published:** 2025-10-07

**Authors:** Guanhua Li, Menghan Chen, Yuchen Lu, Yuxuan Zhang

**Affiliations:** 1 Jiujiang Polytechnic University of Science and Technology, Jiujiang, China; 2 Yantai Research Institute, Harbin Engineering University, Yantai, China; 3 College of Intelligent Science and Engineering, Beijing University of Agriculture, Beijing, China; 4 Department of Computer and Electrical Engineering, Mid Sweden University, Sundsvall, Sweden

**Keywords:** Bearing fault diagnosis, Parallel attention mechanism, Noisy environments, Deep learning, Engineering, Mathematics and computing

## Abstract

In Industry 4.0 intelligent manufacturing, rolling bearings serve as core components of rotating machinery. Their health status directly impacts the safety and reliability of entire manufacturing systems. However, existing fault diagnosis methods face critical challenges in noisy environments, including layer-wise feature information attenuation, insufficient multi-scale feature capture, and limited noise robustness. Such limitations create an urgent need for high-precision and robust deep learning diagnostic techniques. To address these challenges, this study proposes Channel-Time Parallel Attention Network (CT-ParaNet). The network innovatively designs a channel-time parallel attention mechanism that synchronously processes channel and temporal feature correlations to effectively solve information degradation in serial structures. The network constructs multi-scale parallel attention residual blocks using parallel multi-branch architecture with adaptive gating mechanisms to capture and fuse multi-scale fault features. Additionally, it establishes a serial-parallel hybrid processing architecture that systematically integrates parallel attention mechanisms with multi-scale feature extraction modules for hierarchical and parallel fine processing of fault signals. Experimental results on two independent bearing fault datasets show CT-ParaNet achieves accuracies of 98.53% and 98.29%, improving by 15.84 and 16.15% points over traditional methods respectively. Under extreme − 5dB signal-to-noise ratio (SNR) conditions, accuracies remain above 87% across Gaussian white noise, impulse noise, and colored noise environments. With only 0.1 training data ratio, accuracies exceed 92% on both datasets. CT-ParaNet significantly enhances accuracy and robustness of bearing fault diagnosis in noisy environments, providing important technical support for intelligent manufacturing equipment health monitoring.

## Introduction

With the advancement of Industry 4.0, manufacturing is transforming toward intelligent and digital directions^1–4^. Rotating machinery, as core components of industrial production, has made health management a critical element of intelligent manufacturing systems^5^. Prognostics and Health Management (PHM) technology achieves intelligent equipment maintenance through monitoring, diagnosis, and prediction methods^6,7^. Rolling bearings, as essential components of rotating machinery, directly affect the safety and reliability of entire mechanical systems^8^. Therefore, developing high-precision and robust bearing fault diagnosis techniques is crucial for constructing intelligent PHM systems^9,10^.

Traditional fault diagnosis methods in PHM systems primarily rely on expert experience and manual feature extraction, including time-domain statistical analysis, frequency-domain spectral analysis, and time-frequency analysis^11–13^. While these methods perform well under specific conditions, they suffer from limitations such as dependence on professional knowledge for feature extraction, limited model adaptability, and insufficient capability in handling complex operating conditions^14–17^. Deep learning technology has been introduced into mechanical fault diagnosis due to its powerful feature learning and pattern recognition capabilities^18–21^. Deep learning models such as convolutional neural networks^22–24^, recurrent neural networks, and residual networks demonstrate advantages in fault diagnosis tasks through end-to-end processing architectures^25–27^.

However, fault diagnosis in actual industrial environments faces three core challenges. First is complex noise interference, including environmental noise, sensor noise, and internal mechanical system noise, which severely affects the identifiability of fault features^28–30^. Second is the multi-scale characteristics of fault signals, where different fault types exhibit differentiated scale features in time and frequency domains, making it difficult for single-scale feature extraction methods to comprehensively capture essential fault characteristics^31–33^. Additionally, early fault features are typically weak and easily masked by noise, leading to degraded diagnostic performance of traditional deep learning methods in strong noise environments^34–36^.

To address these challenges, existing research primarily adopts three types of solutions. One approach focuses on network architecture improvements by introducing residual connections, attention mechanisms, and multi-scale feature extraction techniques to enhance model noise resistance^37–40^. Another approach emphasizes data augmentation and training strategy optimization by introducing simulated noise during training to improve model generalization capability^41–44^. A third approach adopts strategies combining signal preprocessing with deep learning, first performing denoising on noisy signals before implementing fault identification^45^. However, these methods still have limitations when handling complex industrial noise environments, fundamentally due to four technical deficiencies.

First, traditional serial network architectures suffer from layer-wise feature information attenuation when processing noisy signals. During forward propagation in multi-layer networks, feature transformations at each layer may lead to useful information loss, and this cumulative effect is particularly severe in noisy environments, resulting in insufficient capability for weak fault identification^46–48^. Second, existing attention mechanisms predominantly adopt serial processing strategies, computing channel attention first and then temporal attention^49^, where this sequential dependency easily leads to cumulative loss of critical feature information under noise interference^50–52^. Third, standard convolutional networks have fixed receptive fields, making it difficult to comprehensively capture effective fault information at different scales under complex noise backgrounds, leading to incomplete feature representation. Fourth, existing research lacks unified architectures capable of efficiently integrating different feature extraction modules, limiting overall model noise resistance improvement.

Based on in-depth analysis of the aforementioned technical deficiencies, this study proposes the necessity of parallel processing mechanisms. The information attenuation problem in serial structures requires adopting parallel architectures to avoid cumulative loss during information transmission^53^; the sequential dependency of serial attention mechanisms requires simultaneously capturing feature correlations in channel and temporal dimensions through parallel computation^54^; the limitation of fixed receptive fields requires comprehensively capturing features at different scales through parallel multi-scale branches; the lack of unified architecture requires constructing systematic parallel processing frameworks^55,56^. Therefore, parallel processing is not merely a technical choice but an inevitable requirement for solving core problems in fault diagnosis under noisy environments.

Based on this, this study proposes the CT-ParaNet (Channel-Time Parallel Attention Network) architecture. This architecture provides corresponding solutions to the four technical deficiencies mentioned above:

1. To address the layer-wise information attenuation problem, a channel-time parallel attention mechanism is proposed that synchronously processes feature correlations in channel and temporal dimensions, fundamentally eliminating information attenuation issues in serial structures;
2. To address incomplete feature representation, multi-scale parallel attention residual blocks are designed, employing parallel multi-branch structures with adaptive gating mechanisms to comprehensively capture and fuse multi-scale fault features;
3. To address the lack of unified architecture, a serial-parallel hybrid processing architecture is constructed that systematically integrates parallel attention mechanisms with multi-scale feature extraction modules, achieving hierarchical parallel fine processing of fault signals.

The structure of this paper is organized as follows: Sect. “Proposed method” elaborates on the CT-ParaNet network architecture and the design principles of its core components; Sect. “Experiments and analysis” validates the effectiveness of the proposed method through experimental analysis; Sect. “Conclusion” summarizes the research findings.

## Proposed method

The core challenge in rolling bearing fault diagnosis under noisy environments lies in accurately extracting fault features from noise-contaminated vibration signals. Traditional one-dimensional convolutional neural networks suffer from insufficient feature representation capability when handling noise interference, making it difficult to simultaneously capture temporal sequence dependencies and inter-channel correlations in vibration signals. To address this critical problem, this study proposes an innovative CT-ParaNet channel-time parallel attention network architecture. This network is specifically designed for noisy environments, significantly enhancing the model’s fault identification capability under strong noise interference through parallel processing of attention mechanisms in channel and temporal dimensions, combined with multi-scale feature fusion strategies.

### Problem definition

Rolling bearing fault diagnosis is essentially a multi-classification problem. Given a vibration signal sample set $\mathcal{D}=\{ ({{\mathbf{x}}_i},{y_i})\} _{{i=1}}^{N}$, where ${{\mathbf{x}}_i} \in {{\mathbb{R}}^L}$ represents a one-dimensional vibration signal of length *L*, and ${y_i} \in \{ 1,2, \ldots ,C\}$ represents the corresponding fault category label. This study covers five bearing states: healthy condition, inner race fault, outer race fault, rolling element fault, and cage fault, i.e., $C{\text{ }}={\text{ }}5$.

The goal of fault diagnosis is to learn a mapping function $f:{{\mathbb{R}}^L} \to \{ 1,2, \ldots ,C\}$ to achieve accurate classification of new vibration signals. In actual industrial environments, the observed signal is represented as ${\mathbf{x}}={\mathbf{s}}+{\mathbf{n}}$, where ${\mathbf{s}}$ is the clean signal containing fault information and ${\mathbf{n}}$ is environmental noise. The presence of noise severely affects the identifiability of fault features, requiring diagnostic models to possess strong noise resistance and feature extraction capabilities.

Based on this, the core objective of this study is to design a deep learning architecture specifically for noisy environments, achieving precise fault diagnosis under strong noise interference through innovative attention mechanisms and multi-scale feature processing strategies.

### Adaptive mixing pooling strategy

To address the problem of feature information loss in noisy environments, CT-ParaNet innovatively introduces an adaptive mixing pooling strategy. Traditional pooling operations have inherent defects: maximum pooling can preserve salient features but easily loses weak fault information hidden in noise; average pooling can maintain global information but dilutes the intensity of critical fault features. These limitations are particularly prominent in noisy environments, directly affecting the accuracy of fault diagnosis.

To solve the above problems, this study designs two adaptive mixing pooling mechanisms. Adaptive Mean Mixing Pooling (AMMP) achieves optimal retention of feature information by dynamically balancing the advantages of both pooling operations:

1 $${{\mathbf{U}}_{{\text{AMMP}}}}=\frac{{{{\mathbf{U}}_{{\text{avg}}}}+{{\mathbf{U}}_{{\text{max}}}}}}{2}$$

where ${{\mathbf{U}}_{{\text{avg}}}}$ and ${{\mathbf{U}}_{{\text{max}}}}$ are the output features of average pooling and maximum pooling, respectively. The core principle of this strategy lies in achieving a balance between feature fidelity and robustness through arithmetic averaging.

For scenarios requiring further enhancement of feature discrimination capability, Adaptive Softmax Mixing Pooling (ASMP) employs an exponential weighting mechanism to adaptively emphasize more discriminative features:

2 $${{\mathbf{U}}_{{\text{ASMP}}}}=\frac{{{{\mathbf{U}}_{{\text{avg}}}} \times {e^{{{\mathbf{U}}_{{\text{avg}}}}}}+{{\mathbf{U}}_{{\text{max}}}} \times {e^{{{\mathbf{U}}_{{\text{max}}}}}}}}{{{e^{{{\mathbf{U}}_{{\text{avg}}}}}}+{e^{{{\mathbf{U}}_{{\text{max}}}}}}}}$$

The innovation of ASMP lies in utilizing the nonlinear characteristics of exponential functions to automatically identify and strengthen feature components with higher fault correlation, thereby achieving more precise feature extraction under noise interference.

### Channel-Time parallel attention mechanism

The core innovation of CT-ParaNet lies in the Channel-Time Parallel Attention Mechanism (Channel-Time PAM), which is specifically designed to address the problem of feature correlation being easily disrupted in noisy environments. Traditional attention mechanisms adopt serial processing approaches, computing channel attention first and then processing temporal attention, where this sequential dependency easily leads to cumulative loss of important feature information under noise interference.

Channel-Time PAM fundamentally solves the degradation problem in information transmission by computing attention weights in channel and temporal dimensions in parallel. Its underlying principle is based on feature decoupling and reconstruction: simultaneously projecting input features into channel correlation space and temporal dependency space, independently learning respective attention weights, and finally achieving intelligent feature reconstruction through a learnable fusion MLP.

The channel attention path focuses on identifying correlations between different channels, utilizing adaptive mixing pooling to compress temporal dimension information:

3 $${{\mathbf{X}}_{{\text{Channel}}}}=\operatorname{Sigmoid} (\operatorname{Conv} (\operatorname{AMMP} ({\mathbf{X}})))$$

4 $${{\mathbf{U}}_{{\text{Channel}}}}={{\mathbf{X}}_{{\text{Channel}}}} \odot {\mathbf{X}}$$

The temporal attention path focuses on capturing long-range dependencies in time series, applying the Efficient Channel Attention (ECA) mechanism to learn weight distributions in the temporal dimension after channel dimensionality reduction through 1 × 1 convolution:

5 $${{\mathbf{X}}_{{\text{Time}}}}=\operatorname{Sigmoid} (\operatorname{ECA} ({\text{Conv1}} \times {\text{1}}({\mathbf{X}})))$$

6 $${{\mathbf{U}}_{{\text{Time}}}}={{\mathbf{X}}_{{\text{Time}}}} \odot {\mathbf{X}}$$

The final output of parallel attention is achieved through a learnable fusion MLP:

7 $${{\mathbf{U}}_{{\text{concat}}}}={\text{Concat}}[{{\mathbf{U}}_{{\text{Channel}}}},{{\mathbf{U}}_{{\text{Time}}}}]$$

8 $${{\mathbf{U}}_{{\text{CT-PAM}}}}=\operatorname{MLP} ({{\mathbf{U}}_{{\text{concat}}}}))$$

The key advantage of this parallel architecture lies in that both attention branches act directly on the original input features, avoiding information attenuation in serial processing, while achieving adaptive feature reconstruction through the learnable fusion MLP. Compared to simple additive fusion, this approach can learn more complex channel-temporal feature interaction patterns, significantly enhancing feature representation capability in noisy environments.

### Multi-Scale residual architecture

To address the insufficient capability of single-scale feature extraction in noisy environments, CT-ParaNet employs two innovative multi-scale residual blocks to meet different levels of feature learning requirements.

#### Channel-Time residual block (CT-ResBlock)

CT-ResBlock is the fundamental building unit of CT-ParaNet, with its innovation lying in seamlessly integrating Channel-Time PAM into the residual learning framework. The core idea of this design is to introduce attention mechanisms during the residual mapping process, enabling the network to adaptively strengthen useful features and suppress noise interference:

9 $${\mathbf{H}}({\mathbf{x}})={\mathbf{x}}+\operatorname{CT} -\text{PAM}(F({\mathbf{x}}))$$

where $F({\mathbf{x}})$ represents the residual mapping learned through two convolutional layers, and $\operatorname{CT} -\text{PAM}( \cdot )$ is the channel-time parallel attention operation. The advantage of this structure lies in that the attention mechanism acts directly on the residual branch, ensuring gradient flow stability while enhancing feature discriminative capability.

#### Multi-Scale parallel attention residual block (MSPAR)

The design of the MSPAR is fundamentally rooted in the physical properties of bearing fault signals. Localized defects, such as surface pitting or spalling, generate sharp, transient impacts that manifest as short-term, high-frequency features. In contrast, distributed degradation typically leads to long-term, low-frequency modulation patterns. To simultaneously capture these distinct signal characteristics, the MSPAR block utilizes four parallel branches with varied receptive fields, enabling concurrent extraction of multi-scale information. Critically, the block incorporates a learnable gating mechanism that adaptively adjusts the contribution of each branch based on the input signal. This allows the network to dynamically prioritize the most discriminative feature scales for a specific fault type, thereby creating a robust, data-driven link between the network architecture and the intrinsic physics of the fault signals.

For input features ${\mathbf{x}} \in {{\mathbb{R}}^{B \times C \times L}}$, each branch first extracts multi-scale features in parallel:

10 $${{\mathbf{B}}_i}={\operatorname{Branch} _i}({\mathbf{x}}),\;\;\;{\kern 1pt} i \in \{ 1,2,3,4\}$$

Subsequently, each branch generates adaptive weights through a lightweight gating module:

11 $${g_i}=\operatorname{Sigmoid} (\operatorname{FC} (\operatorname{ReLU} (\operatorname{FC} (\operatorname{GAP} ({{\mathbf{B}}_i})))))$$

where $\operatorname{GAP} ( \cdot )$ is global average pooling and $\operatorname{FC} ( \cdot )$ is the fully connected layer. The gated branch features are:

12 $${\widetilde {{\mathbf{B}}}_i}={g_i} \odot {{\mathbf{B}}_i}$$

Finally, multi-scale feature fusion is achieved through concatenation, attention enhancement, and residual connections:

13 $${{\mathbf{U}}_{{\text{MSPAR}}}}=\operatorname{CT} -\text{PAM}({\text{Concat}}[{\widetilde {{\mathbf{B}}}_1},{\widetilde {{\mathbf{B}}}_2},{\widetilde {{\mathbf{B}}}_3},{\widetilde {{\mathbf{B}}}_4}])+{\mathbf{x}}$$

The four branches have distinct implementation characteristics. The first branch employs a combination of 1 × 1 convolution for dimensionality reduction, AMMP pooling, and 3 × 1 convolution to achieve feature compression and multi-scale enhancement. The second branch adopts the standard combination of 1 × 1 convolution for dimensionality reduction and 3 × 1 convolution to capture medium-scale features. The third branch uses 1 × 1 convolution transformation for channel dimension adjustment rather than direct connection, ensuring feature dimension consistency. The fourth branch achieves larger receptive fields through equivalent implementation of 1 × 1 convolution for dimensionality reduction and 3 × 1 convolution, capturing long-range temporal dependencies. The introduction of the gating mechanism enables the network to dynamically adjust the contribution weights of each branch according to input signal characteristics, significantly improving the robustness and discriminative capability of feature representation in noisy environments.

### Adaptive multi-scale feature fusion

To address the dynamic changes in the importance of different scale features in noisy environments, CT-ParaNet designs an adaptive multi-scale feature fusion mechanism. Traditional fusion methods have inherent limitations. Simple addition assumes equal weights for features at all scales, ignoring the differences in feature importance under different fault modes. Direct concatenation preserves all information but lacks the ability to suppress redundant and noise features.

The adaptive fusion mechanism proposed in this study is based on the idea of learnable weight allocation, achieving intelligent feature reorganization through 1 × 1 convolution. Given multi-scale features from three parallel branches ${{\mathbf{U}}_1} \in {{\mathbb{R}}^{L \times {C_1}}}$, ${{\mathbf{U}}_2} \in {{\mathbb{R}}^{L \times {C_2}}}$, and ${{\mathbf{U}}_3} \in {{\mathbb{R}}^{L \times {C_3}}}$, the fusion process first performs concatenation in the channel dimension:

14 $${{\mathbf{U}}_{{\text{concat}}}}={\text{Concat}}[{{\mathbf{U}}_1},{{\mathbf{U}}_2},{{\mathbf{U}}_3}] \in {{\mathbb{R}}^{L \times {C_{{\text{in}}}}}}$$

where ${C_{{\text{in}}}}={C_1}+{C_2}+{C_3}$. Subsequently, feature reconstruction is performed through adaptive 1 × 1 convolution:

15 $${{\mathbf{U}}_{{\text{fused}}}}(l,k)=\sum\limits_{{m=0}}^{{{C_{{\text{in}}}} - 1}} {{{\mathbf{U}}_{{\text{concat}}}}} (l,m) \times {\mathbf{W}}(m,k)+{\mathbf{b}}(k)$$

The innovation of this mechanism lies in that the weight matrix ${\mathbf{W}}$ and bias vector ${\mathbf{b}}$ are adaptively learned through end-to-end training, enabling dynamic adjustment of contribution weights for features at different scales according to different fault modes and noise conditions, achieving optimal feature representation. Compared to fully connected layers, 1 × 1 convolution maintains spatial locality and significantly reduces computational complexity.

### Overall network architecture

**Fig. 1:**
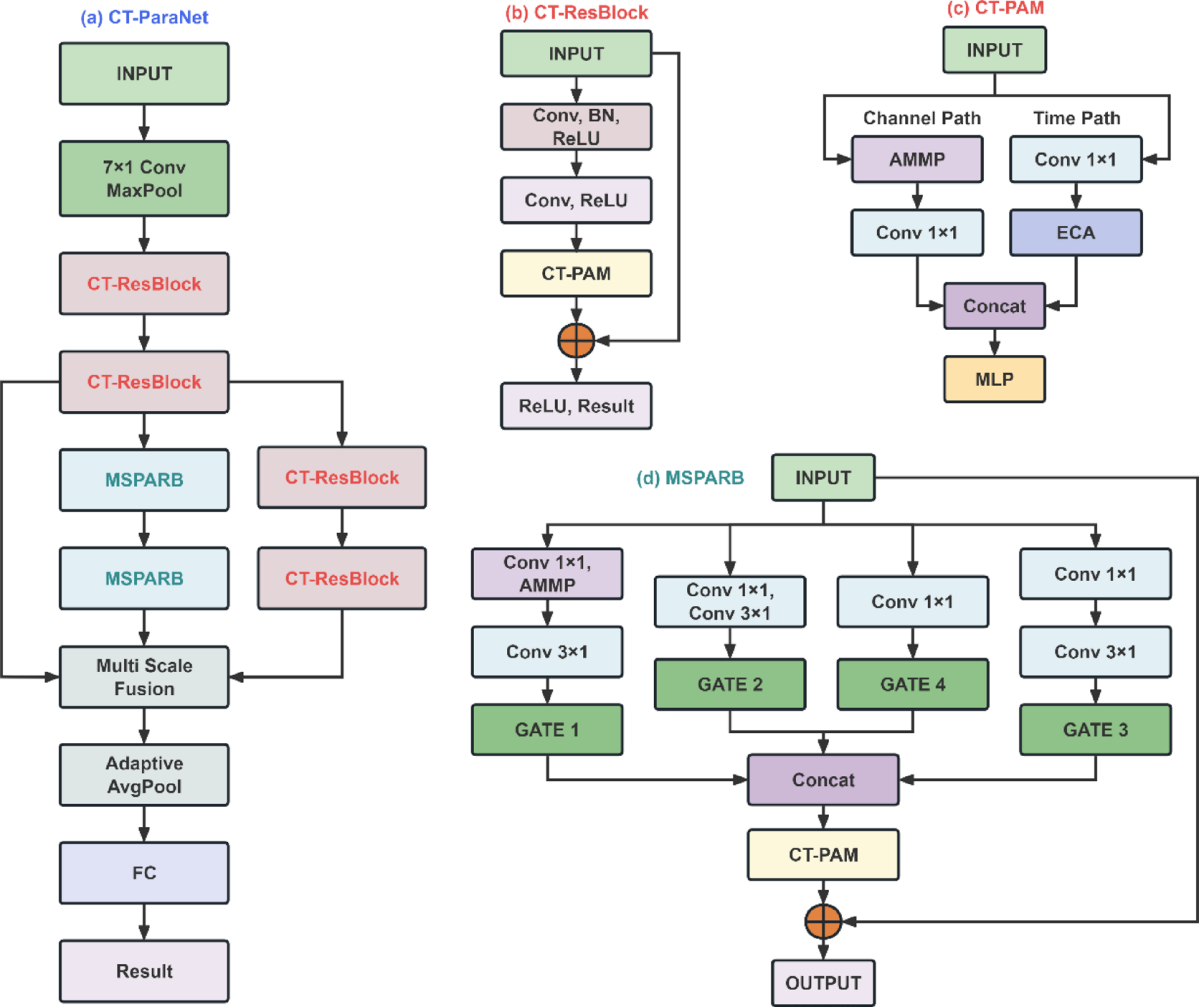
The architectural schematic diagram of CT-ParaNet.

Figure 1 displays the detailed architecture of CT-ParaNet. Overall, CT-ParaNet adopts an end-to-end hierarchical feature learning architecture, specifically optimized for fault diagnosis tasks in noisy environments. The overall architecture follows a hierarchical design philosophy from coarse-grained feature extraction to serial-parallel hybrid multi-scale processing, then to adaptive fusion and final classification decisions.

The initial feature extraction stage employs 7 × 1 convolutional kernels with stride 2 for coarse-grained feature mapping, a design that reduces computational complexity while preserving key fault features. Subsequently, batch normalization and ReLU activation ensure training stability, and 3 × 1 max pooling is applied for further dimensionality reduction, laying the foundation for subsequent multi-scale processing.

After feature extraction, the network employs a serial-parallel hybrid structure for multi-scale processing:

The first branch serves as the basic feature path, employing CT-ResBlock to construct standard residual connections, focusing on learning basic fault feature representations, with its output serving as the input source for subsequent branches.

The second branch serves as the multi-scale enhancement path, taking the output of the first branch as input, implementing multi-receptive field feature extraction based on CT-MSPAR blocks, simultaneously capturing short-term impact features and long-term modulation features.

The third branch serves as the long-range dependency path, also taking the output of the first branch as input, employing CT-ResBlock with large 5 × 1 and 7 × 1 convolutional kernels to specifically capture long-range temporal dependencies in bearing fault signals.

The outputs of the three branches are intelligently integrated through the adaptive multi-scale feature fusion mechanism, then compressed into fixed-length feature vectors through global average pooling, and finally achieve fault classification through fully connected layers.

CT-ParaNet’s core advantages lie in several key aspects. First, the hybrid serial-parallel structure balances basic and advanced feature learning. The initial serial path serves as the backbone, stably extracting low-level features, which are then passed to parallel branches designed to capture multi-scale and long-range dependency features. This hybrid approach avoids the issue of feature dilution seen in purely parallel architectures while leveraging parallel processing for efficient and comprehensive multi-scale feature fusion, systematically enhancing the model’s representational power. Second, the multi-scale design ensures comprehensive coverage of different fault modes. Third, the adaptive fusion mechanism enables dynamic optimization of feature weights. Finally, end-to-end training ensures the collaborative optimization of all modules, achieving outstanding fault diagnosis performance even in noisy environments.

## Experiments and analysis

### Experimental datasets

**Fig. 2:**
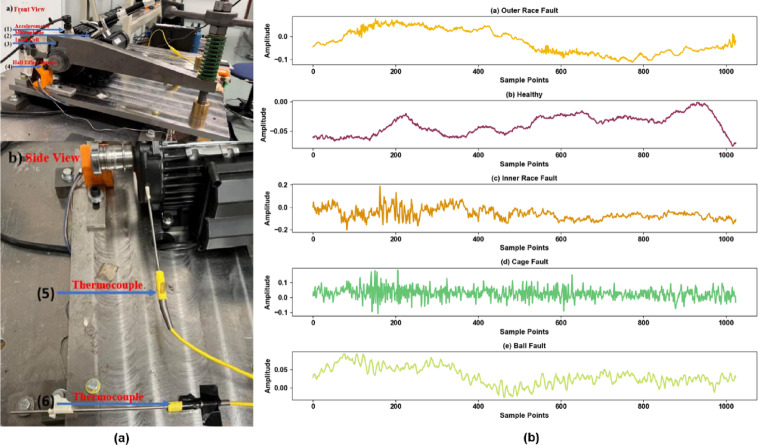
**(a)** University of Ottawa experimental setup for bearing fault diagnosis. **(b)** Example vibration signals recorded from the setup.

Dataset A originates from the University of Ottawa bearing fault diagnosis dataset, with data collected through a specially designed UORED-VAFCLS experimental platform under constant speed conditions of 1750 RPM. This dataset employs accelerometer sensors, with a data acquisition sampling frequency of 200 kHz, to record bearing vibration signals. It covers five bearing operating states: healthy condition, inner race fault, outer race fault, rolling element fault, and cage fault, with each state corresponding to an independent time-series signal channel. The experimental setup of Dataset A and signal examples collected under different bearing states are shown in Fig. 2.

**Fig. 3:**
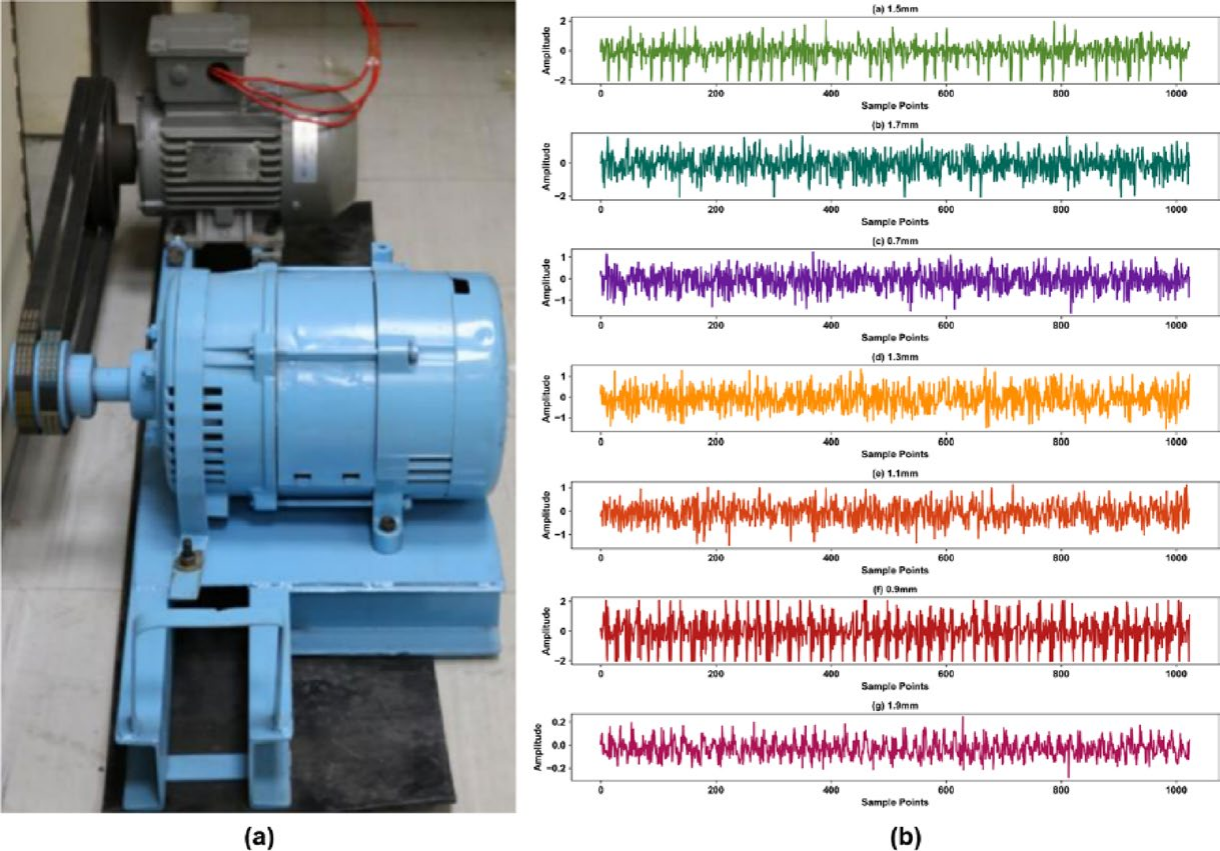
**(a)** Mehran University of Engineering and Technology (MUET) experimental setup for bearing fault diagnosis. **(b)** Example vibration signals recorded from the setup.

Dataset B is released by the NCRA Condition Monitoring Systems Laboratory at Mehran University of Engineering and Technology, Pakistan, as a three-phase induction motor bearing vibration dataset. This study selects a data subset under 100 W constant load conditions, which contains six different damage sizes for bearing outer race faults. These faults, with dimensions of 0.7 mm, 0.9 mm, 1.1 mm, 1.3 mm, 1.5 mm, and 1.7 mm, were artificially introduced using electrical discharge machining (EDM), and their precise sizes were physically verified post-creation to ensure data label accuracy. The experimental setup of Dataset B and signal examples collected under different bearing damage states are shown in Fig. 3.

To ensure the reliability and fairness of experimental results, both datasets employ the same preprocessing pipeline. Raw vibration signals are segmented using sliding window technique with a window length of 1024 sampling points and a step size of 512 sampling points. The window length of 1024 was chosen to ensure that each sample contains sufficient data to capture at least one full rotation cycle of the bearing, which is critical for identifying the periodic impact features characteristic of most bearing faults, while also maintaining a manageable input size for the deep learning model. Each signal segment is automatically labeled with fault categories according to its source. To avoid the influence of data partition bias on results, all experiments adopt an 8:2 ratio for random division of training and test sets, with stratified sampling performed to maintain consistent proportions of each fault class. This process is independently repeated 5 times, with each experiment conducted on a new random partition basis, and final results are averaged over 5 experiments, ensuring evaluation objectivity and statistical significance.

### Experimental settings and model configuration

To evaluate the effectiveness of CT-ParaNet, six representative methods were selected as benchmarks: a multi-scale CNN with channel attention (CA-MCNN)^57^, mixed information CNN (MIXCNN)^58^, wide kernel CNN (WDCNN)^59^, LSTM, ResNet18, and Transformer. These methods reflect key paradigms in fault diagnosis: WDCNN and ResNet18 represent classical and deep convolutional networks, CA-MCNN combines attention with a multi-scale framework, MIXCNN emphasizes lightweight design, and LSTM demonstrates the strengths of recurrent networks in sequence modeling, Transformer, on the other hand, utilises a self-attention mechanism that can effectively capture long-distance dependencies and is suitable for dealing with complex fault characteristics. This selection provides a systematic and fair baseline for comparison.

All comparative methods adopt identical training configurations as CT-ParaNet to ensure comparability of experimental results. The training process employs the Adam optimizer with an initial learning rate of 0.001 and a batch size of 32. All models are trained for 100 epochs using cross-entropy loss function for optimization. The training environment is based on the PyTorch framework with accelerated computation on NVIDIA GPUs. To ensure result stability and reproducibility, each experiment is independently repeated 5 times, with final results averaged and standard deviations calculated.

The proposed CT-ParaNet adopts an end-to-end hierarchical feature learning architecture, with detailed parameter configurations shown in Table 1. CT-ParaNet is designed to achieve robust fault feature extraction and precise classification in noisy environments through channel-time parallel attention mechanisms and multi-scale residual structures.

**Table 1:** Proposed CT-ParaNet model configuration.

Component	Parameter Configuration
Input Layer	7 × 1 Conv with Stride 2 and 64 Channels, Max Pooling with 3 × 1 and Stride 2
Branch 1 CT-ResBlock	Channel progression from 64 to 64 to 128, Kernel size 3 × 1
Branch 2 CT-MSPARBlock	Channel progression from 128 to 128 to 256, Four parallel multi-scale branches
Branch 3 CT-ResBlock	Channel progression from 128 to 128 to 256, Kernel sizes 5 × 1 and 7 × 1
Feature Fusion	1 × 1 Conv from 640 input channels to 256 output channels
Classifier	Global Average Pooling with Fully Connected Layer from 256 to Num Classes
General Configurations	ReLU Activation, Batch Normalization, Channel-Time PAM with AMMP Pooling

### Evaluation metrics

To comprehensively and fairly evaluate the diagnostic capability of models in noisy environments, this study selects accuracy and F1-score as the primary evaluation metrics. First, accuracy is used to reflect the proportion of overall classification correctness of the model, defined as follows:

16 $${\text{Acc}}=\frac{{TP+TN}}{{TP+TN+FP+FN}}$$

Meanwhile, to comprehensively consider both classification precision and recall capability, this study adopts the F1-score as a supplementary metric, which is defined as the harmonic mean of precision and recall:

17 $${\text{F1}}=\frac{{2 \times {\text{Precision}} \times {\text{Recall}}}}{{{\text{Precision}}+{\text{Recall}}}}$$

where: $${\text{Precision}}=\frac{{TP}}{{TP+FP}}$$,$$\;{\kern 1pt} {\text{Recall}}=\frac{{TP}}{{TP+FN}}$$.

In multi-class classification tasks, this paper adopts macro-averaged F1, which calculates F1 for each class separately and then takes the arithmetic mean, ensuring consistent weight for each class and improving sensitivity to minority classes.

### Comparison with existing methods

To comprehensively evaluate the diagnostic performance of CT-ParaNet, this study conducts comparative experiments with five representative deep learning methods on two datasets. Table 2 presents detailed comparative results, where CT-ParaNet achieves optimal performance on all evaluation metrics, fully validating the effectiveness of the proposed method.

**Table 2:** Performance comparison of different methods on two datasets.

Method	Dataset A			Dataset B	
	Accuracy		F1-score	Accuracy	F1-score
CA-MCNN	0.9126		0.9114	0.8773	0.8688
MIXCNN	0.9585		0.9581	0.9239	0.9248
WDCNN	0.9530		0.9528	0.9594	0.9548
LSTM	0.8369		0.8058	0.8214	0.8215
ResNet18	0.9774		0.9773	0.9674	0.9670
Transformer	0.9512		0.9498	0.9442	0.9412
**CT-ParaNet**	**0.9853**		**0.9853**	**0.9829**	**0.9830**

Experimental results demonstrate that CT-ParaNet achieves significant performance improvements on both datasets. On Dataset A, accuracy reaches 98.53%, representing a 0.79% point improvement over the second-best ResNet18. On Dataset B, accuracy achieves 98.29%, with an improvement of 1.55% points. F1-scores also demonstrate excellent performance, reaching 98.53% and 98.30% respectively.

Detailed comparative analysis reveals significant performance differences among methods. LSTM performs worst on both datasets, with accuracies of 83.69% and 82.14% respectively, primarily limited by insufficient feature learning capability due to gradient vanishing problems. Although CA-MCNN introduces multi-scale convolution and channel attention, its effectiveness remains relatively poor. MIXCNN, as a lightweight model, employs depthwise separable convolution design to improve computational efficiency, but still exhibits deficiencies in complex fault feature extraction. The wide convolutional kernel design of WDCNN and the Transformer model, which excels at capturing global dependencies, both perform well on two datasets. However, there is still a certain gap in their performance when compared to CT-ParaNet.

In summary, CT-ParaNet’s superior performance stems from its innovative architectural design: the channel-time parallel attention mechanism simultaneously captures multi-dimensional feature correlations while avoiding information loss in serial processing; multi-scale residual structures achieve comprehensive feature extraction; adaptive fusion mechanisms optimize feature weight allocation. The synergistic effect of these designs enables CT-ParaNet to demonstrate significant performance advantages in complex mechanical fault diagnosis tasks.

### Performance in noisy environments

To validate the robustness of CT-ParaNet in actual industrial environments, this study designs systematic noise robustness experiments. Various noise interferences in industrial sites severely affect bearing fault feature identification. This study selects three typical noise types: Gaussian white noise simulates sensor circuit noise and environmental random interference; impulse noise simulates mechanical impacts and gear meshing transient interference, easily confused with bearing fault impact characteristics; pink noise has power spectral density inversely proportional to frequency, simulating specific spectral interference such as low-frequency vibrations and structural resonance in bearing systems. By controlling signal-to-noise ratio from − 5dB to 30dB, the diagnostic capability of CT-ParaNet under different noise intensities is systematically evaluated.

**Fig. 4:**
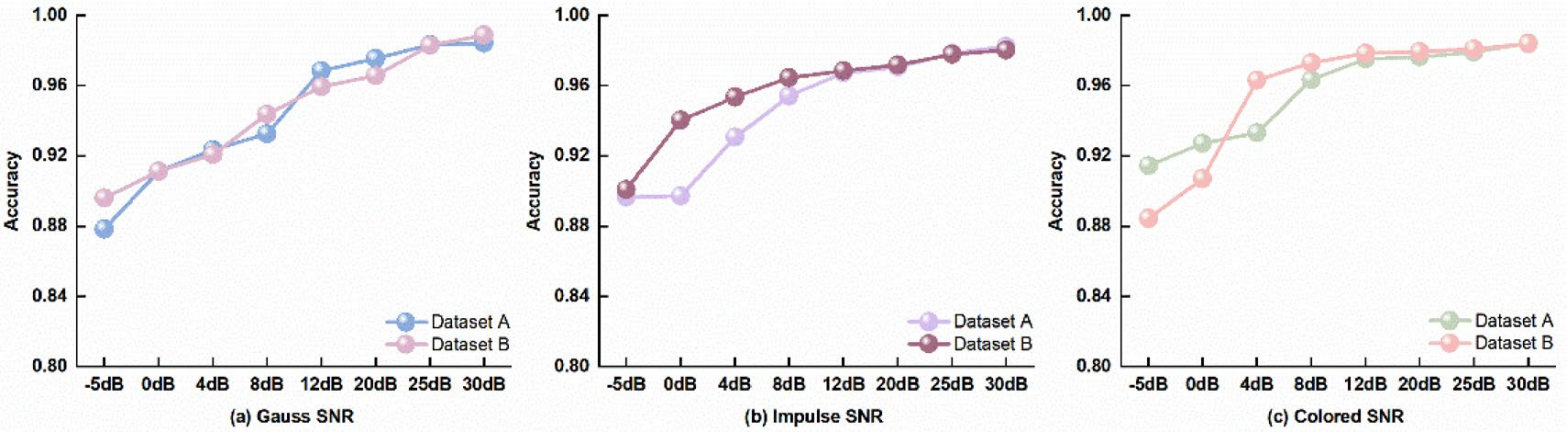
Performance of CT-ParaNet under different noise conditions: **(a)** Gaussian white noise, **(b)** Impulse noise, **(c)** Pink noise.

Figure 4 demonstrates the performance of CT-ParaNet under three noise environments. As the signal-to-noise ratio increases, model accuracy steadily rises on both datasets, demonstrating excellent noise resistance capability.

Under Gaussian white noise environments, CT-ParaNet demonstrates excellent robustness. Under extremely low signal-to-noise ratio conditions of −5dB, Dataset A and Dataset B still achieve accuracies of 87.85% and 89.60%, significantly exceeding traditional methods’ performance in clean environments. When the signal-to-noise ratio increases to 30dB, accuracies rise to 98.46% and 98.91%, approaching ideal performance, indicating that CT-ParaNet can effectively suppress sensor circuit noise interference.

Impulse noise testing further validates CT-ParaNet’s anti-interference capability. Under extreme − 5dB conditions, accuracies on both datasets reach 89.68% and 90.10% respectively, demonstrating greater stability than in Gaussian noise environments. This proves that the CT-PAM mechanism can effectively distinguish bearing fault impacts from other interference impacts in mechanical systems, maintaining the integrity of true fault features. At high signal-to-noise ratio of 30dB, accuracies reach 98.26% and 98.05%.

Test results under pink noise environments are most prominent. CT-ParaNet steadily improves from 91.45% at −5dB to 98.45% at 30dB on Dataset A, and from 88.45% to 98.42% on Dataset B. The model performs better under low signal-to-noise ratio pink noise conditions than other noise types, indicating that the multi-scale residual architecture can effectively handle specific spectral noise interference such as low-frequency vibrations and structural resonance in bearing systems.

Comprehensive analysis demonstrates that CT-ParaNet exhibits excellent robustness under all noise types and intensities. Its superior noise resistance performance stems from three key designs: the channel-time parallel attention mechanism adaptively suppresses noise features while strengthening fault information; multi-scale residual structures capture weak fault features in noise through different receptive fields; adaptive mixing pooling strategies preserve critical information while filtering out noise interference. The synergistic effect of these innovative designs enables CT-ParaNet to maintain high-precision fault diagnosis capability even under extreme noise environments.

### Performance with varying training data ratios

In practical industrial applications, obtaining large quantities of labeled fault samples often faces challenges of high cost and time constraints. To evaluate the learning capability of CT-ParaNet under limited training sample conditions, this study designs experiments with varying training data ratios. The experiments select training set ratios from 0.1 to 0.4, systematically evaluating model diagnostic performance under different training sample scales.

**Fig. 5:**
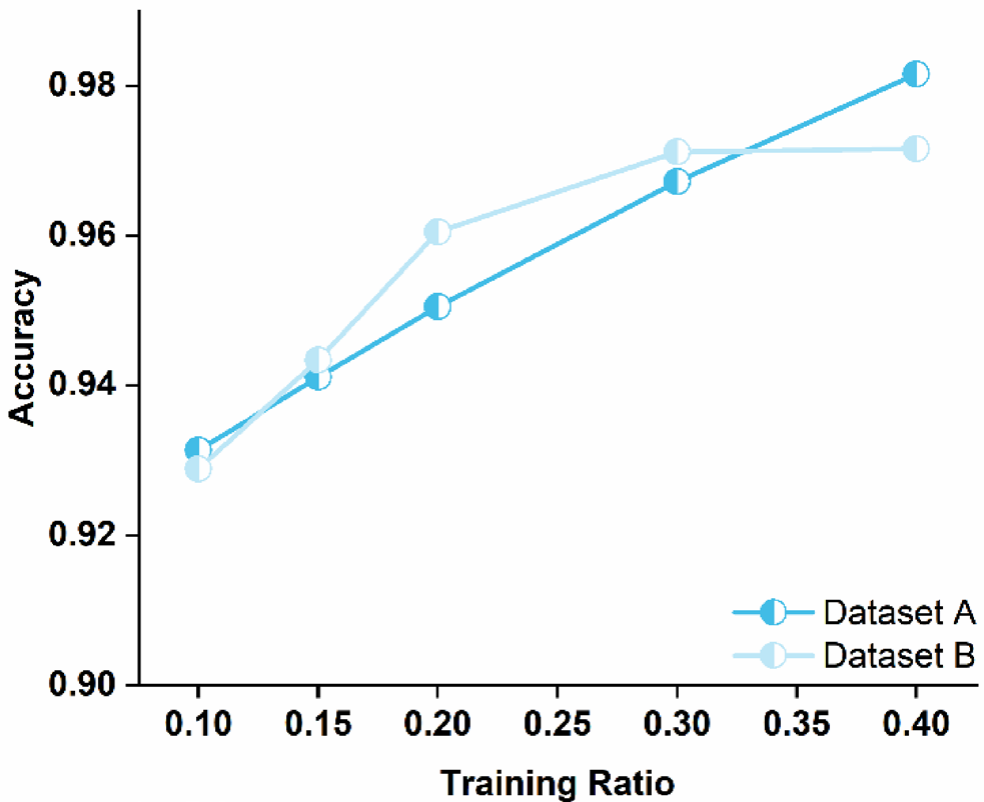
Performance of CT-ParaNet with varying training data ratios on Dataset A and Dataset B.

Figure 5 demonstrates the performance variation trends of CT-ParaNet under different training data ratios. Experimental results indicate that CT-ParaNet can still maintain high diagnostic accuracy under limited training sample conditions.

Experimental results on Dataset A show that when the training ratio is 0.1, CT-ParaNet’s diagnostic accuracy is 93.14%. As the training data ratio increases to 0.15 and 0.2, accuracies improve to 94.11% and 95.05% respectively. When the training ratio reaches 0.3, accuracy further rises to 96.72%. Under a 0.4 training ratio, accuracy reaches 98.16%.

Experimental results on Dataset B exhibit similar trends. Under 0.1 training ratio conditions, CT-ParaNet achieves 92.89% accuracy. When the training ratio increases to 0.15, accuracy rises to 94.34%. When training ratios are 0.2 and 0.3, accuracies reach 96.05% and 97.12% respectively. Under a 0.4 training ratio, accuracy is 97.16%.

From the experimental results on both datasets, it can be observed that CT-ParaNet’s diagnostic performance exhibits a stable upward trend as the training data ratio increases. Even under relatively limited training sample conditions with a 0.1 ratio, the model achieves accuracies exceeding 92% on both datasets, indicating that the proposed network architecture possesses good feature learning capability. The channel-time parallel attention mechanism can extract effective discriminative features from limited samples by simultaneously processing feature correlations in channel and temporal dimensions. Multi-scale residual structures improve the model’s utilization efficiency of limited training data through feature extraction with different receptive fields. These designs enable CT-ParaNet to maintain stable diagnostic performance under training sample-constrained conditions.

### Training strategy analysis

To further validate the robustness of CT-ParaNet’s architectural design, this study conducts systematic analysis of the impact of different optimization strategies on model performance. Five mainstream optimization algorithms are selected: Adam, AdamW, SGD, RMSprop, and Adagrad, evaluating the influence of different optimization strategies on CT-ParaNet’s diagnostic performance under identical network configurations and training settings.

**Fig. 6:**
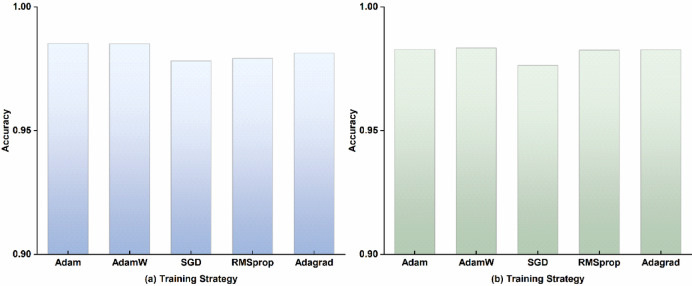
Performance comparison of CT-ParaNet with different optimization strategies: **(a)** Dataset A, **(b)** Dataset B.

Figure 6 presents the performance comparison results of five optimization algorithms on both datasets. Experimental results demonstrate that CT-ParaNet exhibits good robustness to different training strategies, with all optimization algorithms enabling the model to achieve high diagnostic accuracy.

Experimental results on Dataset A show that performance differences among the five optimization algorithms are relatively small. The Adam optimizer achieves the best performance with an accuracy of 98.53%. AdamW follows closely with an accuracy of 98.51%, only 0.02% points lower than Adam. Adagrad achieves 98.14% accuracy, RMSprop reaches 97.92%, and SGD performs relatively worse but still attains a high accuracy of 97.82%. The gap between the highest and lowest accuracies is only 0.71% points, indicating that CT-ParaNet is insensitive to optimization strategy selection.

Experimental results on Dataset B further validate this conclusion. AdamW leads slightly with an accuracy of 98.34%, while Adam achieves 98.29%, with only a 0.05% point difference between them. RMSprop and Adagrad achieve accuracies of 98.26% and 98.27% respectively. Although SGD performs relatively worse, its accuracy still reaches 97.64%. All five optimization algorithms maintain accuracies above 97.6%, with a maximum difference of only 0.7% points.

Comprehensive experimental results from both datasets indicate that CT-ParaNet’s superior performance primarily stems from its innovative network architecture design rather than specific optimization strategy selection. The synergistic effect of core components including channel-time parallel attention mechanisms, multi-scale residual structures, and adaptive feature fusion enables the model to stably converge to high-performance states under different optimization algorithms. This characteristic holds significant importance for practical industrial applications, allowing users to flexibly select optimization strategies according to specific requirements and computational resources without concerns about significant performance loss.

### Limitations of the CT-ParaNet

While CT-ParaNet demonstrates significant advantages in accuracy and noise robustness, several limitations must be acknowledged to guide future research. First, the complex parallel and multi-scale architecture, while effective, introduces higher computational costs compared to simpler, lightweight models, which may pose challenges for real-time monitoring on resource-constrained edge devices. Second, the current study validated the model’s performance under constant speed and load conditions, but its adaptability to highly variable operating conditions, particularly the challenges in industrial applications such as wind turbines, requires further exploration. Finally, although the model was tested under three typical noise types, real industrial noise is typically a complex, non-stationary mixture of multi-source interference, which poses greater challenges than the controlled environments used in this study.

## Conclusion

In the context of rapid intelligent manufacturing development, high-precision mechanical fault diagnosis technology has become crucial support for ensuring production safety and improving manufacturing efficiency. Addressing core challenges in rolling bearing fault diagnosis under noisy environments, including layer-wise feature information attenuation, incomplete multi-scale feature capture, and limited noise resistance performance, this study proposes the CT-ParaNet channel-time parallel attention network architecture. This architecture avoids information attenuation in serial structures through parallel attention mechanisms, comprehensively captures complex fault features using multi-scale parallel attention residual blocks, and constructs serial-parallel hybrid processing architectures to enhance overall noise resistance performance. Experimental validation demonstrates that CT-ParaNet achieves excellent diagnostic performance on two independent datasets, with accuracies reaching 98.53% and 98.29% respectively, representing improvements of at least 15.84 and 16.15% points over traditional methods, demonstrating significant performance advantages. In noise robustness testing, even under extreme − 5dB signal-to-noise ratio conditions, the proposed method maintains accuracies above 87% across three typical noise environments, fully validating its reliability in harsh industrial environments. Furthermore, CT-ParaNet maintains excellent performance under limited training sample conditions, with accuracies exceeding 92% on both datasets when the training ratio is only 0.1, demonstrating good sample utilization efficiency. Training strategy analysis further confirms the robustness of the network architecture, achieving stable convergence to high-performance states under five different optimization algorithms, providing flexible configuration choices for practical industrial deployment. CT-ParaNet provides important technological breakthroughs for intelligent manufacturing equipment health monitoring, with its innovative parallel attention mechanism significantly improving fault diagnosis accuracy in noisy environments, laying a technical foundation for Industry 4.0 intelligent production system construction.

From an industrial perspective, CT-ParaNet demonstrates strong noise robustness, enabling adaptation to complex operating conditions such as variable speed and heavy loads in wind turbine gearboxes, as well as the high precision requirements of CNC machine tool spindles. Its end-to-end capability reduces reliance on handcrafted feature engineering and domain expertise, thereby facilitating deployment. Nevertheless, transitioning to real-world applications remains challenging. Real-time diagnostics impose strict constraints on computational resources and inference speed, which calls for further network optimization. In addition, industrial environments often involve multi-source mixed noise, typically arising from the superposition of Gaussian noise, impulse noise, and harmonic interference, which is considerably more complex than the single-noise cases tested in this study. future research will focus on the following directions: first, exploring model compression and quantization techniques to develop a lightweight version of CT-ParaNet suitable for embedded applications; second, integrating domain adaptation and transfer learning strategies to enhance diagnostic performance under variable operating conditions; and third, constructing more complex and realistic mixed-noise datasets to further validate and improve the model’s robustness for industrial deployment.

## Data Availability

The datasets used and/or analyzed during the current study are available from the corresponding author on reasonable request.
